# Phylogenetic Diversity of *Trichoderma* Strains and Their Antagonistic Potential against Soil-Borne Pathogens under Stress Conditions

**DOI:** 10.3390/biology9080189

**Published:** 2020-07-23

**Authors:** Omar A. Hewedy, Khalid S. Abdel Lateif, Mahmoud F. Seleiman, Ashwag Shami, Fawziah M. Albarakaty, Rasha M. El-Meihy

**Affiliations:** 1 Department of Plant Agriculture, University of Guelph, 50 Stone Road East, Guelph, ON N1G 2W1, Canada; 2 Department of Genetics, Faculty of Agriculture, Menoufia University, Shibin El-Kom 32514, Egypt; k_dein2001@yahoo.com; 3 Department of Biotechnology, College of Science, Taif University, Taif 21944, Saudi Arabia; 4 Plant Production Department, College of Food and Agriculture Sciences, King Saud University, P.O. Box 2460, Riyadh 11451, Saudi Arabia; 5 Department of Crop Sciences, Faculty of Agriculture, Menoufia University, Shibin El-kom 32514, Egypt; 6 Biology Department, College of Sciences, Princess Nourah bint Abdulrahman University, Riyadh 11617, Saudi Arabia; AYShami@pnu.edu.sa (A.S.); fmbarakati@uqu.edu.sa (F.M.A.); 7 Department of Biology, College of Applied Sciences, Umm AlQura University, Makkah Al Moukarramh 21955, Saudi Arabia; 8 Department of Agricultural Microbiology, Faculty of Agriculture, Benha University, Moshtohor 13736, Egypt; 9 School of Chemistry, Chemical Engineering and Life Science, Wuhan University of Technology, Wuhan 430070, China

**Keywords:** *Trichoderma*, antagonism, RAPD, SSR, *TEF1* sequencing, phosphate solubilization, diversity

## Abstract

*Trichoderma* species are known as excellent biocontrol agents against soil-borne pathogens that cause considerable crop losses. Eight strains of *Trichoderma* were isolated from five Egyptian regions. They identified based on translation elongation factor-1α (*TEF1*) sequencing as four different *Trichoderma* species: *Trichoderma asperellum*, *Trichoderma harzianum*, *Trichoderma viride*, and *Trichoderma longibrachiatum*. Optimal growth conditions (temperature and media), and the phosphate solubilization capability of *Trichoderma* strains were evaluated in vitro. Further, the ability of these strains to antagonize *Fusarium solani*, *Macrophomina phaseolina*, and *Fusarium graminearum* was also evaluated. The results revealed that *Trichoderma harzianum* (*Th6*) exhibited the highest antagonistic ability against *F. solani*, *M. phaseolina* and *F. graminearum* with inhibition rates of 71.42%, 72.97%, and 84.61%, respectively. *Trichoderma viride* (*Tv8*) exhibited the lowest antagonism against the same pathogens with inhibition rates of 50%, 64% and 69.23%, respectively. Simple-sequence repeats (SSRs) and random amplified polymorphic DNA (RAPD) markers were used to evaluate the genetic variability of the *Trichoderma* strains. The results revealed that of 45 RAPD amplified bands, 36 bands (80%) were polymorphic and of SSRs amplified 36 bands, 31 bands (86.11%) were polymorphic. The amplification of calmodulin and *β-1,3-endoglucanase* was noted at 500 bp and 230 bp, respectively. Data indicated that *T. viride* (*Tv8*) had the highest phosphate solubilization index (10.0 mm), while *T. harzianum* (*Th6*) had the lowest phosphate solubilization index (4.0 mm). In conclusion, *T. harzianum* (*Th6*) had the highest antagonistic activity in dual culture assay along with the growth rate; while *T. viride* (*Tv8*) had the highest phosphate solubilization activity. There are still gaps in obtaining new formulations, selecting potent *Trichoderma* strains to confirm disease control in planta. For improving *Trichoderma* recommendation in the organic agricultural system and sustaining the fertility of the soil, the field application of highly antagonistic biocontrol agents in different types of soil and plant species will be the first approach toward bio-pesticide treatments along with bio-fertilizer inoculation. Furthermore, secondary metabolites will be investigated for the most promising strains with the combination of different pathogens and application timing.

## 1. Introduction

Plant health and crop production are constantly under threat from biotic constraints, especially fungal diseases caused by various species of soil fungal pathogens. Even though farmers believe that chemical fungicides can control these diseases, some already use them in combination biological control agents. These are of great attention as pesticides have unfavorable side impacts, and it is necessary to find an alternative method such as beneficial microbes to prevent human and environmental disasters. *Trichoderma* is a soil fungal genus of great economic importance which is ubiquitous under various climatic conditions [1] and has the ability to live in soil stress conditions for instance salinity, alkalinity, nutrient deficiency and drought [2]. Several *Trichoderma* species are beneficial to host plants, providing one of the most promising alternatives for promoting plant growth [3] by increasing the plant health and its immunity, motivating mechanisms of plant defense, avoiding pathogen outbreaks and controlling plant disease [4,5]. In particular, *T. harzianum* [6]; *T. asperellum* [7]; *T. asperelloides* [8]; *T. virdie* [9], and *T. longibrachiatum* [10] are promising biological control agents against soil-borne pathogens that suppress plant health. *Trichoderma* spp. plays an essential role through pathogens inhibition under the contact zone due to its multiple biocontrol mechanisms such as mycoparasitism, antibiosis, competition for sites and nutrients [11], availability of metals [12], non-volatile and volatile compounds production [13], extracellular hydrolytic enzymes production [7], and pathogen’s enzymes inactivation [14]. Biotic and abiotic factors influence antagonistic activity and growth of *Trichoderma* species against pathogens, including growth medium [6,15], temperature [16] and metal availability [17]. *Trichoderma* strains use several modes of action, including acidification, chelation, reduction, and hydrolysis to regulate the availability of metals such as phosphate and iron solubilization. Although *Trichoderma* can live under different environmental conditions, temperature plays a substantial function in enhancing its growth and mycoparasitic activity [18,19,20]. The harmful pathogenic fungi that *Trichoderma* can inhibit include, *Fusarium solani* [21], *F. graminearum* [22], and *Macrophomina phaseolina* [23]. Understanding biodiversity is important for determining biological resources and sustainable development.

Theoretically, morphological taxonomy of *Trichoderma* is primarily based on macro. and micro-morphological characteristics which includes colony color, shape, size, appearance on specific culture media, and spore-forming structures. However, the greatest reliable method to identify an unidentified isolate at the taxon level by molecular approaches is by phylogenetic analysis [24]. This molecular identification is important for the precise identification of *Trichoderma* species, and it is challenging to distinguish *Trichoderma* fungi by traditional identification methods using only morphological characterization [25,26]. To discover diverse *Trichoderma* species, it is imperative to have reliable phylogenetic information, and more precise molecular methods have substituted traditional identification approaches in most taxonomic investigation. The internal transcribed spacer (ITS) is considered the universal fungal molecular barcode, but it cannot distinguish numerous closely interrelated species and has low species resolution in the genus *Trichoderma* [24]. Taxonomic *Trichoderma* communities need to develop specific practices for molecular characterization in their identification. Partial translation elongation factor 1-alpha (*TEF1*) sequences have been suggested for the phylogenetic analysis of such genus to assess the accuracy and robustness of DNA barcoding and to discover previously undescribed species [27].

Various authors have reported that *TEF1* has a high phylogenetic efficacy because of: (i) it is more relative to the identification of unknown species; and (ii) it is alienable among *Trichoderma* species [28,29,30].

Not surprisingly, the sequencing of ITS and/or *TEF1* has been extensively studied [10,31,32,33], reference databases make it possible to submit DNA sequences of environmental isolates and link sequence data to phenotypic data. In particular, molecular DNA markers are employed as a reliable tool to determine the genetic diversity and relationships [34,35]. RAPD is one of the valuable markers which has been utilized to determine the biodiversity of *Trichoderma* species, due to its use in identifying mysterious genomes and its requirement of only limited quantities of DNA [36,37,38,39,40,41]. SSR has likewise been proved to be a robust marker to investigate polymorphism and discriminate between different species of *Trichoderma* [42,43,44].

Another important gene encodes calmodulin, which plays a pivotal role in the antifungal ability of *Trichoderma* and its hyphal growth rate, serving as one of the critical antagonistic mechanisms to compete with fungal pathogens. Calmodulin can be involved in the formation of germinative tubes, and play a role in fungal growth, developmental structures, and differentiation [45].The expression of many genes is regulated by certain physiological events, i.e., growth on different media (with glucose) or by starving the cells of different amino acids. These regulatory proteins are a complex system and involve a variety of regulated proteins.

Furthermore, they depend on the function of signal transduction pathways to link between the extracellular signal such as nutrients, environmental conditions and the transcriptional response expression [46]. One of these regulatory proteins was studied in this investigation by amplification of calmodulin (*cal*) gene to prove the linkage between the abiotic stress (nutrients and temperature) and the viability of growth rate and sporulation among *Trichoderma* strains under these stressful conditions. The presence of calmodulin has been detected in *Trichoderma viride*. It affected the vegetative growth and starvation-induced conidiation of *T. viride* significantly and it is necessary for both fungal dimorphism and mycelial growth [47].

The aims of this investigation were to evaluate the genetic diversity among eight strains of *Trichoderma* obtained from different geographic locations of Egypt based on RAPD and SSR markers and their antigenic activity against soil-borne pathogens under stress conditions to gain a better understanding of how *Trichoderma* spp. enhance its defense systems under stressful conditions. Besides, phosphate solubilization capability of *Trichoderma* strains were investigated.

## 2. Materials and Methods

This research consisted of eight experiments conducted in 2019. The molecular work was conducted at the Laboratory of Biotechnology, Horticulture Research Institute, Agricultural Research Center, Giza, Egypt. The antifungal activity, along with phosphate solubilizing experiments, were carried out at the Department of Agricultural Microbiology. Faculty of Agriculture, Benha University, Egypt.

### 2.1. Trichoderma Strains

Eight *Trichoderma* strains previously isolated from different Egyptian locations and identified using the internal transcribed spacer (ITS) [10] were used in this study. These strains were belonged to four species, including *T. asperlum* (*Ta*), *T. harzianum* (*Th*), *T. longibrachiatum* (*Tl*), and *T. viride* (*Tv*); coded as described in [10].

### 2.2. Pathogenic Fungi

Three pathogenic fungi, *Fusarium solani*, *Macrophomina phaseolina*, and *Fusarium graminearum* were grown on potato dextrose agar (PDA) at 28 °C, then maintained at 4 °C for further experiments. These pathogens were obtained from the Plant Pathology Research Institute, ARC, Egypt.

### 2.3. TEF1 Sequencing for Identification of Trichoderma Strains

The present work was derived from previous phylogenetic analysis and identification of 15 strains [10]. To confirm the previous ITS identification of the fungal strains, the *TEF1* region was amplified using two primers (Eurofins) named *EF1-728F* (5′CATCGAGAAGTTCGAGAAGG3′) and *TEF1 R* (5′GCCATCCTTGGAGATACCAGC3′) as previously described [48,49].

PCR reactions of the *TEF1* region were amplified in a total volume of 20 μL reaction mixture; 0.5 μL each of both forward and reverse primers (10 pmol/μL), 1.0 μL of purified DNA (10 ng/μL), 10 μL of 2 × PCR Master Mix buffer and 8 μL of ultrapure sterile water by Applied Biosystems™ SimpliAmp™ Thermal Cycler (Catalog No.A24811). Detection of PCR products was carried out by loading 5 μL of each sample onto a 1.8% agarose gel alongside a GeneRuler 100 bp DNA Ladder (catalog no.SM0241). The PCR products were Sanger-sequenced by the Big-Dye Terminator v3.1 sequencing kit in a total volume of 20 μL with a 3730xl automated sequencer (Applied Biosystems, Foster City, CA, USA). Nucleotide sequences were determined on both strands of the PCR amplification products at the Macrogen sequencing facility (Macrogen Inc., Seoul, Korea). The phylogenetic tree of the *TEF1* gene was then built using MEGA version 7 software to show the similarity of *Trichoderma* strains. The sequences of the *TEF1* fragments were analyzed using the nucleotide BLASTn program, and the National Center for Biotechnology Information (NCBI) database was used to test for similarity with known *Trichoderma* strains in combination with TrichOKey v. 2.0 http://www.isth.info/ [50].

### 2.4. Evaluation of the Genetic Diversity of Trichoderma Strains

To study the phylogenetic relationship between the eight antifungal strains, seven sets of PCR primers for each of two different molecular markers (SSR [42,44], RAPD) was used as shown in Table 1.

#### 2.4.1. RAPD Analysis of *Trichoderma* Strains

All the fungal species were cultured on 50 mL of PDA at 28 °C for 96–120 h in the dark. Then, Genomic DNA was extracted from the collected fungal mycelium using a spin column method (Qiamp mini kit, Qiagen GmbH, Hilden, Germany) with slight modifications as previously described [51]. The PCR amplification reaction of the purified DNA samples was performed in a total volume of 25 μL mixture as previously described [52]. The amplification reaction was carried out in a thermal cycler (catalog no. A24811) programmed with a slight modification of the amplification cycles; 5 min at 94 °C for the initial denaturation followed by 35 cycles, each consisting of a denaturation step of 1 min at 94 °C, an annealing step for 1 min at 32–36 °C depending on the primers, and an elongation step for 2 min at 72 °C with a final extension for 10 min at 72 °C. Amplified PCR products were separated by electrophoresis with 2% agarose gels containing ethidium bromide and visualized under ultraviolet light.

#### 2.4.2. SSR Analysis of *Trichoderma* Strains

The SSR amplification reactions were conducted in a total volume of 50 μL; 25 ng of DNA template, 1 × PCR buffer, 25 μL MyTaq™ Red Mix, 8 μL DNA Template, 1 μL each of the forward and reverse primers (10 pmol/μL), and 15 μL nuclease-free water. For the SSR markers, PCR conditions began with an initial denaturation for 3 min at 95 °C, followed by 35 cycles of denaturation for 15 s at 94 °C, annealing for 45s at 52–56 °C depending on the primers, and the extension for 1 min at 72 °C, with a final extension for 10 min at 72 °C. These PCR products were migrated in 1.5% agarose gel in 0.5 × TBE buffer at 90 V for 90 min with a 1000 bp DNA ladder as a size marker. The gel was stained with ethidium bromide (0.5 μg/mL) and visualized under a gel documentation system.

### 2.5. Specific Trichoderma Genes

#### 2.5.1. Calmodulin (*cal*) Gene

Based on the highest and lowest growth rate of different *Trichoderma* strains, four strains (*Ta1, Th4, Th6 and Tv8*) were used to detect (*cal*) gene for testing the optimal growth conditions (temperature and media). Calmodulin gene (*cal*) was amplified according to previously published protocol [53].

#### 2.5.2. β-1,3-endoglucanase

The primer design and PCR amplification of β-1, 3-endoglucanase were performed as previously described [54].

### 2.6. Activities of Trichoderma Strains under Stress Conditions

#### 2.6.1. Growth under Thermal Stress and Different Substrates

In this experiment, the ability of *Trichoderma* strains to grow at different temperatures below and above the optimum temperature was estimated using two incubation temperatures (25 °C and 35 °C) along with two different growth media: PDA and cornmeal dextrose agar (CMD) [55]. PDA was composed of infusion from 200.0 g/L potatoes, 20.0 g/L dextrose, and 15.0 g/L agar, with a final pH of 5.6 ± 0.2, whereas CMD was composed of infusion from 50.0 g/L cornmeal, 2.0 g/L dextrose, and 15.0 g/L agar, with a final pH of 6.0 ± 0.2.

For each strain, a 5-mm disk was separately inoculated at the centre of 90-mm Petri dishes containing 10 mL of each medium. The number of replications for each treatment was three. All Petri dishes were divided into two groups; the first was incubated at 25 °C and the second was incubated at 35 °C. Mycelial growth was recorded after seven days [14].

#### 2.6.2. Ability to Solubilize Insoluble Phosphate

The assay was modified from previously described method [8,56] to test the ability of *Trichoderma* strains for phosphate solubilization. Briefly, each strain was singly cultured on a Petri dish (100 mm) containing 10 mL of NBRIP agar composed of 10.0 g/L glucose, 5.0 g/L MgCl_2_·6 H_2_O, 0.25 g/L MgSO_4_·7H_2_O, 0.2 g/L KCl, 0.002 g/L FeSO_4_·7H_2_O, 0.5 (NH_4_)_2_SO_4_, 0.5 g/L yeast extract, 10.0 g/L tri-calcium phosphate (Ca_3_HPO_4_), and 15.0 g/L Bacto-agar, dissolved in 950 mL distilled water with the pH adjusted to 7.2. Glucose was separately filter sterilized and mixed with autoclaved medium. The dishes were inoculated with a 5-mm fungal disk, then incubated at 28 ± 0.2 °C for seven days. Then, *Trichoderma* plates were examined for a clear zone around the colonies. The diameter of these P solubilization zones was measured and calculated by applying the formula for phosphate solubilization index (PSI) [57].

(1) $$\mathrm{PSI}=\frac{\mathrm{colony}\;\mathrm{diameter}+\mathrm{halo}\;\mathrm{zone}\;\mathrm{diameter}}{\mathrm{colony}\;\mathrm{diameter}}$$

#### 2.6.3. The Antifungal Ability of *Trichoderma* Strains against Soil-Borne Pathogenic Fungi

*Trichoderma* strains were evaluated against three soil-borne pathogens; *Fusarium solani*, *Macrophomina phaseolina*, and *Fusarium graminearum* by a dual culture technique as previously published [58]. Briefly, 5-mm diameter mycelial disks from the edge of 7 days-old cultures of *Trichoderma* fungi and the soil-borne pathogens were simultaneously cultured on the opposite of the plate at an equal distance from the margin. The experimental design was completely randomized with four replications for each strain along with the control plates (which had no *Trichoderma*). Inoculated Petri dishes (90 mm) were incubated at 28 °C. The antagonistic activity was estimated after 5–7 days of incubation by measuring the radius (mm) of the pathogen colony in the control plate (R1) and the radius of the pathogen colony in the direction of the antagonist colony (R2). The inhibition percent of mycelial growth (PIMG) was calculated formulas described by [59,60]:

(2) $$\mathrm{PIMG}\;\left(\%\right)=\frac{R1-R2}{R1}\times 100$$

where: R1 = radial growth of pathogen in control plate, R2 = radial growth of pathogen in dual culture plate.

#### 2.6.4. Rate and Speed of Growth

All *Trichoderma* strains and *F. graminearum* were grown at the same time on PDA and observed daily to record their growth.

### 2.7. T. harzianum (Th6) against F. graminearum

#### 2.7.1. Mycoparasitic Activity at Different Temperatures

This experiment was separated into three groups; the first was incubated at 4 °C, the second at 28 °C, and the last at 37 °C. All Petri dishes were incubated for five days and checked daily for mycoparasitic activity.

#### 2.7.2. Slide Culture Method

The *T. harzianum* (*Th6*) and *F. graminearum* interaction was visualized using light microscopy (Olympus BX61, Tallahassee, FL, USA) as the procedure described [61]. Then, each *Trichoderma*–pathogen hyphae contact was observed on a thin PDA film on the slide. The hyphae interactive zones were cut into pieces at different growth rates, which were then observed directly under a light microscope for the presence of coiling structures form wall disintegration.

### 2.8. Statistical Analysis

Data were statistically analyzed using analysis of variance and comparisons of means at a 5% level of significance with a Duncan’s multiple range test analysis performed with one-way analysis of variance using IBM SPSS Statistics v25.0 software. Amplicons were scored for their presence (1) or absence (0) in each strain. Cluster analysis of the binary data was performed using the NTSYS-pc v.2.1 program [62]. Similarity matrices were generated using Jaccard’s coefficients, and an unweighted pair-group method using arithmetic averages (UPGMA) was chosen to generate the dendrogram from the RAPD and SSR similarity matrices.

## 3. Results and Discussion

### 3.1. Molecular Experiments

#### 3.1.1. *TEF1* Gene for Confirming the Identification of *Trichoderma* Strains

The molecular identification of strains based on the previously described ITS sequences [10] and the fragment of the *TEF1* gene confirmed that all of the strains used in this study belong to four *Trichoderma* species.

**Figure 1:**
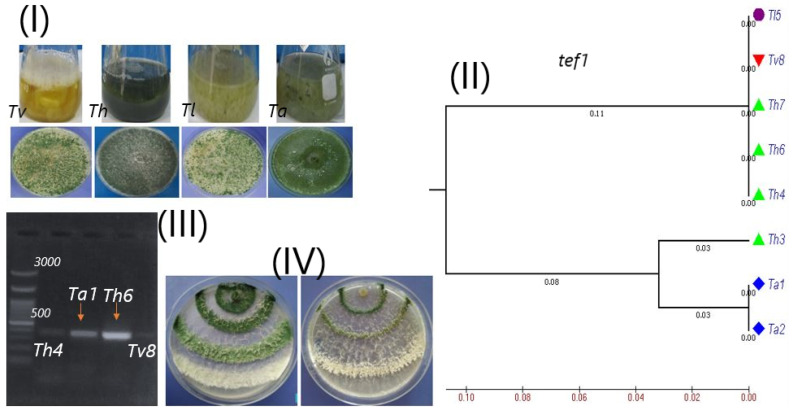
(**I**) *Trichoderma* species used in this study. (**II**) Phylogenetic tree of the identified isolates based on the *TEF1* sequences dataset. The numbers below the branches indicate bootstrap values. (**III**) Calmodulin gene (*cal*) detection in the strains with the highest (*Ta1*, *Th6*) and the lowest (*Th4*, *Tv8*) growth rate test. (**IV**) The growth rate of *Trichoderma* strains on different growth media. *Ta1* = *Trichoderma asperlum*; *Ta2* = *Trichoderma asperlum*; *Th3* = *Trichoderma harzianum*; *Th4* = *Trichoderma harzianum*; *Tl5* = *Trichoderma longibrachiatum*; *Th6* = *Trichoderma harzianum*; *Th7* = *Trichoderma harzianum*; *Tv8* = *Trichoderma viride*.

PCR amplification of the *TEF1* region gave one band of approximately 610 bp. The PCR products were sequenced and, the data compared with the reference data on NCBI using BLASTn. A tree containing eight strains of *Trichoderma* was drawn; the tested strains were found to be closed to four taxa of *Trichoderma* belonging to *T. harzianum* (*Th3*, *Th4*, *Th6* and *Th7*), two strains were classified as *T. asperellum* (*Ta1* and *Ta2*), one strain was identified as *T. longibrachiatum* (*Tl5*), and, one strain belonged to *T. viride* (*Tv8*) (Figure 1). The phylogenetic tree of *Trichoderma* species based on *TEF1* sequences divided the strains into two major clusters. The first cluster included the strains *Th4*, *Th6*, *Th7*, *Tv8* and *Tl5*, while the second cluster contained the strains *Ta1*, *Ta2* and *Th3* (Figure 1). The diversity of the phylogenetic analysis between ITS and *TEF1* markers is understandable as each marker targets different genomic regions. A previously study [63] investigated the biodiversity of *Trichoderma* spp. using both ITS and *TEF1* regions and found that *TEF1* has enough variations to discriminate between different species of *Trichoderma*. Our results agree with previous studies, highlighting the effectiveness of *TEF1* marker for the identification and discrimination of different *Trichoderma* species [31,32,33].

The amplification of two specific genes (*cal* and *β-1,3-endoglucanase*) was noted at 500 bp and 230 bp, respectively (Figure 1III). In agreement with previous studies, β-1,3-endoglucanase is an important gene with an essential role in encoding cell wall degrading enzymes (CWDE) and is induced by metabolites secreted by *Trichoderma* under biotic stress. Furthermore, it plays a vital role in the mycoparasitic activity against the pathogens, especially soil-borne pathogens, after the interaction contact [64,65,66].

Considering the other important gene, the calmodulin signaling pathway plays an essential role in the conidiation and hyphal growth of *Trichoderma* fungi. It might be considered the main cytoskeleton component for regulation of nuclear transcription factors that affect the expression level of other genes.

The diversity of *Trichoderma* strains is noted to include different species that can vary from each other in their phenotypic characterization, including growth rate, conidium morphology and biogeography. Molecular analysis utilizing fragments of calmodulin genes indicates phenotypic characteristics typical of *Trichoderma* strains.

A previous study [67] concluded that external Ca^2+^ induced growth-independent cellulase production, hyphal growth, and total protein secretion of *T. reesei* Rut-C30 via the (Ca^2+^/calmodulin) signal transduction pathway. Thus, our results might be used for more efficient chitinase and β-1,3-endoglucanase enzymes production by *T. asperellum* (*Ta1*) and *T. harzianum* (*Th6*), as well as providing a suitable approach to understand the regulatory mechanisms due to environmental interactions.

#### 3.1.2. RAPD Analysis of *Trichoderma* Strains

**Figure 2:**
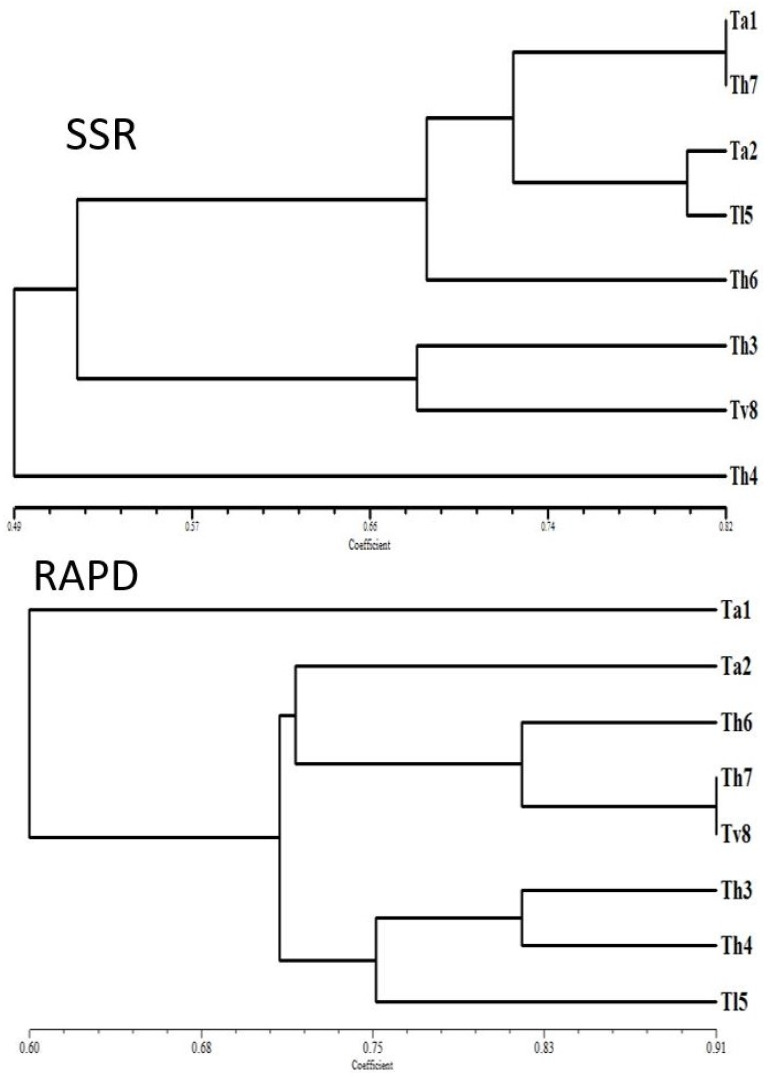
Dendrogram based on Jaccard’s similarity coefficients scored from RAPD and SSR data using the UPGMA algorithm representing 8 *Trichoderma* strains (*Ta1*, *Ta2*, *Th3*, *Th4*, *Th6*, *Th7*, *Tl5* and *Tv8*). *Ta1* = *Trichoderma asperlum*; *Ta2* = *Trichoderma asperlum*; *Th3* = *Trichoderma harzianum*; *Th4* = *Trichoderma harzianum*; *Tl5* = *Trichoderma longibrachiatum*; *Th6* = *Trichoderma harzianum*; *Th7* = *Trichoderma harzianum*; *Tv8* = *Trichoderma viride*.

A set of seven decamer RAPD primers was used for further evaluation of genetic diversity of the eight Egyptian *Trichoderma* strains (*Ta1*, *Ta2*, *Th3*, *Th4*, *Tl5*, *Th6*, *Th7* and *Tv8*). A total of 45 bands were obtained by PCR; 36 (80%) of these produced polymorphic patterns among tested strains (Table 2). The number of polymorphic bands ranged from four (OP-A3, OPA04 and OP-B9) to eight (OPA05). The highest similarity value (0.91) was found between isolates *Th7* and *Tv8*, while the lowest similarity value (0.53) was found between two pairs of isolates (*Ta1* and *Th3*; *Ta1* and *Th6*). RAPD amplicons were scored for their absence and presence, and 36 bands differentiating strains were used to build a binary matrix, and then a dendrogram showing genetic diversity (Figure 2). The cluster analysis divided the strains into two groups; the first group contained the strains (*Ta2*, *Th3*, *Th4*, *Tl5*, *Th6*, *Th7* and *Tv8*) while, the second group contained only the strain *Ta1*. Interestingly, the strains *Th3*, *Th4*, *Th6* and *Th7* were found in the same group, while, the strains of *Ta1* and *Ta2* were classified into separate groups. These results are consistent with previous work [68] which studied the polymorphisms of cultivated peanut genotypes using the SCoT marker and found that not all strains related to the same variety were classified in the same group. In addition, another study [39] indicated that the RAPD marker revealed a genetic diversity among *T*. *asperellum* isolates. In our work, the UPGMA dendrogram illustrated that *T. asperellum* isolates could not be grouped by their lytic enzymes production and/or antifungal activity. Previous studies employed the RAPD marker to evaluate the genetic relationships between different *Trichoderma* isolates and the results indicated high level of polymorphisms [39,40,41,69,70].

#### 3.1.3. SSR Analysis of *Trichoderma* Strains

A total of 36 bands were detected, out of which 31 (86%) were polymorphic bands (Table 2). The number of polymorphic bands for each strain was three (SR8) to six (TvCAT32). The highest value of similarity (0.82) was noted between strains *Th7* and *Ta1*. In contrast, the lowest similarity value (0.35) was found between the strains *Th3* and *Th7* (Table 2). The cluster analysis divided the strains into two main groups; the first group contained the strains (*Ta1*, *Ta2*, *Th3*, *Tl5*, *Th6*, *Th7* and *Tv8*) while, the second group contained only the strain *Th4*. Interestingly, all the strains of *T. asperillum* and *T. harizianum* were found together in the same cluster. In general, SSR markers exhibited more polymorphism than RAPD markers and both markers established specific genetic relationships between strains. The variability in the polymorphisms and cluster analysis obtained with SSR and RAPD markers is understandable since each marker amplifies different genomic regions. An earlier study [42] investigated the genetic variability among different *Trichoderma* strains using SSRs and RAPD markers and found that more polymorphisms are obtained with SSRs (>77%) than with RAPD (~50%). Moreover, another study [43] compared the occurrence of SSRs in *T. atroviride*, *T. harzianum*, *T. reesei*, and *T. virens* and revealed that the occurrence, abundance, and density of microsatellites differed among the different species of *Trichoderma*. This highlights the efficacy of SSR markers in establishing new genetic relationships between the different strains of *Trichoderma*.

### 3.2. Activities of Trichoderma Strains under Stress Conditions

#### 3.2.1. Growth under Thermal Stress and Different Substrates

*Trichoderma* growth relies on the balance between nutrients and substrates to promote conidiation, although identification of the optimal growth conditions requires further research. Of the physical parameters, temperature plays the most critical role in enhancing fungal growth. In addition to temperature, a previous study reported that the carbon and nitrogen (C:N) ratio influences conidiation in *Trichoderma* growth [71].

In this experiment, *Trichoderma* strains were grown on two growth media (PDA and CMD) and incubated at two temperatures to estimate their ability to grow under thermal stress. The data presented in Table 3 indicate that all tested *Trichoderma* strains were able to grow on different media but at various growth rates. The *Trichoderma* strains showed a distinct variation in their phenotypic characteristics depending on the culture media, with a clear difference in the morphological growth patterns between PDA and CMD media. The growth rate at 25 ºC for all strains except for *Tv8* and *Th3* was higher on PDA than CMD except for *Th6*. At 25 ºC on PDA, *Ta1* and *Tv8* had the highest and lowest growth rates, *respectively*, and on CMD, *Th6* and *Th4* had the highest and lowest growth rates, respectively. Several studies have claimed that potato dextrose medium is the best choice for the growth of *Trichoderma* fungi [6,14,15,72]. One study [73] evaluated the growth of *T. harzianum* on five culture media namely, PDA, modified potato dextrose agar, water agar (WA), carrot agar (CA) and cornmeal agar (CMA), PDA and WA were found to be more and less effective for growth, respectively. In another study, wheat bran was found to be the best medium out of four media tested, including PDA, for *Trichoderma* spp. growth [74].

On the other hand, at 35 ºC, the growth rate for all strains except for *Th3* and *Th4* strains was higher on PDA than CMD. The data in Table 3 show that *Th6* had the highest growth rate on both media, and that *Th4* and *Tv8* had the lowest growth rate on PDA and CMD, respectively. This might be due to the different *components* in the two media and the potato extract in PDA that provide the essential elements for fungi growth. Moreover, 25 ºC was the best incubation temperature for all *Trichoderma* strains regardless of the medium used for growth. Similar results by another group [75] proved that 25 °C promotes more mycelial growth of *T. harzianum* than 15 °C. Another group, Ref [19] observed an increase in the mycelial growth of all *Trichoderma* isolates at temperatures ranging from 12 ºC to 27 ºC, and then decreased up to 37 ºC, being inhibited at 42 ºC.

Moreover, another study [18] showed that all *Trichoderma* species grew at different temperatures 20 °C, 25 °C, 30 °C, and 35 °C but grew best at a temperature range of 25 °C to 30 °C. Similarly, Ref [76] proved that *T. viride* grew at temperatures ranging from 10 °C to 30 °C, with maximum growth at 25 °C. In addition, 25 °C was found to be the optimum incubation temperature for *T. harzianum* [6], while its highest antagonistic *potential* was at 20 °C and the optimum range for the growth of *T. harzianum* and *T. viride* was 20–30 °C [77].

#### 3.2.2. Ability to Solubilize Insoluble Phosphate

**Figure 3:**
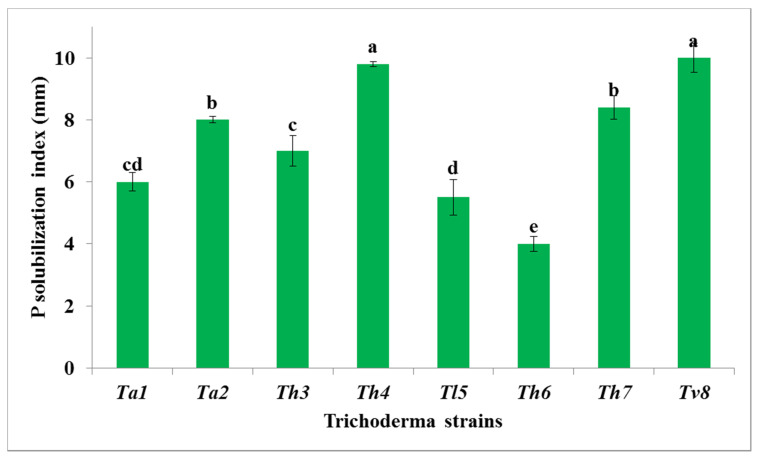
Growth of *Trichoderma* species on Modified Pikovskaya’s Agar medium (MPA) supplemented with Rock Phosphate (RP) for phosphate -solubilization after seven days’ incubation at 28 ± 0.2 °C. *Ta1* = *Trichoderma asperlum*; *Ta2* = *Trichoderma asperlum*; *Th3* = *Trichoderma harzianum*; *Th4* = *Trichoderma harzianum*; *Tl5* = *Trichoderma longibrachiatum*; *Th6* = *Trichoderma harzianum*; *Th7* = *Trichoderma harzianum*; *Tv8* = *Trichoderma viride*.

**Figure 4:**
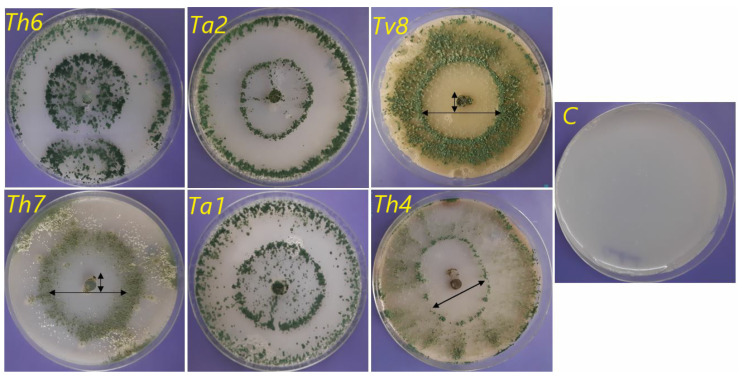
Growth of *Trichoderma* species on Modified Pikovskaya’s Agar medium (MPA) supplemented with Rock Phosphate (RP) for phosphate -solubilization after seven days’ incubation at 28 ± 0.2 °C.

Solubilization of elements by biocontrol agents is achieved by chelation, reduction and hydrolysis. These *mechanisms* play a role in their effective biocontrol activity under various environmental conditions. The results were illustrated in Figure 3 and Figure 4 and indicated that *T. viride* (*Tv8*) had the highest phosphate solubilization activity, followed by *T. harzianum* (*Th4*). On the other hand, *T. harzianum* (*Th6*) had the lowest phosphate solubilization ability. Our results proved that both *T. asperellum* strains (*Ta1* and *Ta2*) had a higher phosphate solubilization index (6 and 8 mm, respectively) as per a previous report [78] that *T. asperellum* Q1 was able to produce phosphatase enzymes for phosphate solubilization under salt stress conditions. Similar results were also recorded in another study [8], which found that *T. asperelloides* and *T. harzianum* were the highest phosphate solubilizers of five strains studied and suggested that *Trichoderma* strains used the solubilized phosphate in their cellular processes. These results were logical as it has been reported [79] that fungi have a large capability for solubilizing rock phosphate.

The variation of clear zones around the fungal growth shown in Figure 4 might be due to their ability to produce organic acids that can reduce pH and enhance the phosphates solubilisation as well as some of *micro- and macro-nutrients* [12,80,81]. Moreover, one study [1] reported that *T. koningiopsis* could solubilize phosphate under stress conditions such as alkalinity and drought by producing organic acids, accumulated polyphosphate in its mycelia, and produced alkaline phosphatase enzyme. One paper [82] suggested three possible mechanisms for phosphate solubilization by *Trichoderma* species—acidification of the microenvironment, production of chelating compounds, and redox activity- but, this paper denied the secretion of organic acids by *Trichoderma* strains, namely oxalic, citric, DL-malic, succinic, DL-lactic, and fumaric acids. However, another group [83] investigated these organic acids in two *T. harzianum* strains and their relationship with the promotion of tomato plant growth. Moreover, the recent study by Hewedy et al. [10], reported that the application of different *Trichoderma* strains significantly improved the growth parameters of pepper plants. A further study, [84] pointed out that phosphate can be solubilized in the absence of detectable chelating agents or organic acids but can be done by acidification of the medium. Finally, *Trichoderma* strains should have a higher stress tolerance than pathogens, qualifying them to carry out their work as biological control agents [85].

#### 3.2.3. Ability to Antagonize Soil-Borne Pathogenic Fungi

**Figure 5:**
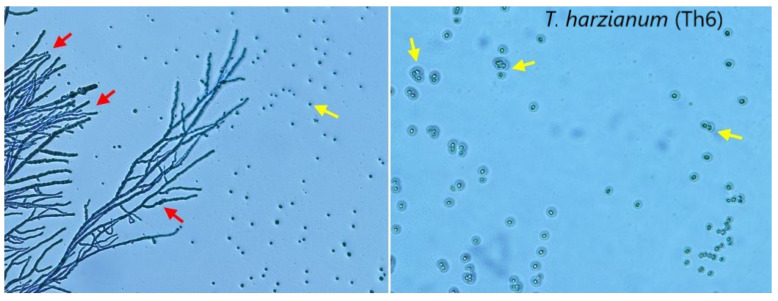
The conidiophores (red arrows) and conidia (yellow arrows) of the *Th6* strain.

**Figure 6:**
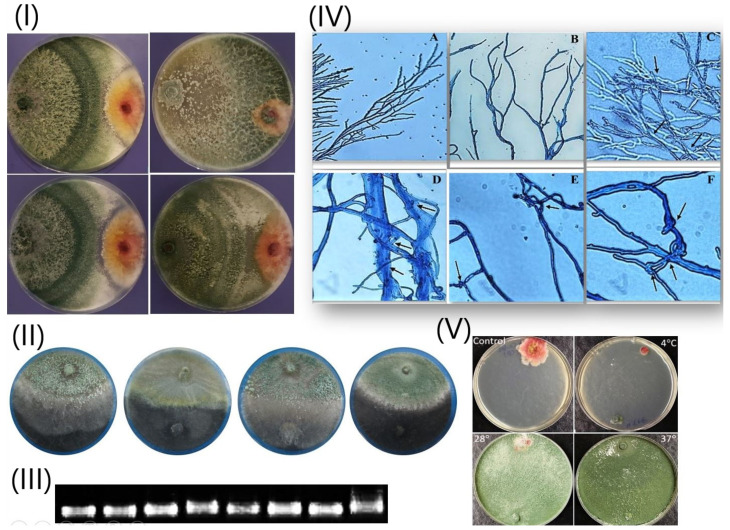
(**I**) In vitro dual culture assay of different *Trichoderma* strains against *F. graminearum*; (**II**) In vitro dual culture assay of different *Trichoderma* strains against *Macrophomina phaseolina*; (**III**) Detection of β-1,3-endoglucanase gene for eight *Trichoderma* strains; (**IV**) Examination of the confrontation activity of *Th6* strain and *Fg* using light microscopy: (A) *T. harzianum* (*Th6*) as control without pathogen; (B) *F. graminearum* (*Fg*) as control without *Trichoderma*; (C–F) the interaction between *Th6* and *Fg* at a different time: (C) 10 min; (D) 20 min; (E) 30 min and (F) 60 min. The arrows mean the mycoparasitism processes involving the hyphal interactions by the attachment and coiling (dark blue) in the panel D and E before killing the pathogen in panel F. (**V**) Growth inhibition of pathogenic fungus (*Fg*) mycelia in vitro dual culture with *Th6* at different temperatures (4 °C, 28 °C and 37 °C).

Plant pathogenic fungi cause harmful effects and the control of plant diseases by chemical pesticides represent a global problem. Moreover, the high cost linked with the only use of fungicides to reduce the development of plant disease, caused by soil-borne fungi, is not an effective approach. Thus, antifungal activity and the capability of various Egyptian *Trichoderma* strains to inhibit the growth of three soil-borne pathogens were tested in this work, as shown in (Table 4). The inhibition percentage of radial growth of *F. solani* in dual cultures was observed among *Trichoderma* strains, and *T.* *harzianum* (*Th6*; Figure 5) had the highest antagonistic activity. All *Trichoderma* strains consistently inhibited *M. phaseolina* as they grew superficially along with its colony and inhibited its growth by percentage ranged from 64.05% to 72.97% (Figure 6I,II). Extreme levels of inhibition were found in the *F. graminearum*–*Trichoderma* interactions, with inhibition percentage ranging from 69.23% to 84.61%. These strains were recently tested against *F. oxysporum f.* sp. *Capsici* under greenhouse conditions, the results showed that the isolates Th7 and Th6 were the most effective *Trichoderma* strains in the suppression of Disease severity (DS) in plants foliage with 68.47% and 65.77% of reduction, respectively. Furthermore, this study revealed that *Th7* was the most effective *Trichoderma* isolate in the suppression of DS in plants foliage (68.47% of reduction) followed by *Th6* (65.77% of reduction) while the *Th3* isolate was the lowest [10]. Inhibition of pathogens growth in the contact zone with *Trichoderma* spp. in dual cultures might be attributed to the production of inhibitory volatile and non-volatile compounds such as terpenes, pyrones, and polyketides [13], production of extracellular hydrolytic enzymes [7], and inactivation of the pathogen’s enzymes [14].

Generally, the data indicated that *T.* *harzianum* (*Th6*, Figure 5) caused the highest inhibitor for all pathogenic strains. *F. graminearum* was the weakest pathogenic strain, showing inhibition of 84.61% by *Th6*. In contrast, *T. viride* (*Tv8*) had the lowest inhibition percent for all pathogenic strains. Similar results were previously recorded by [15] in a study which tested *T. harzianum* against three pathogenic fungi (*Phyllosticta sphaeropsoidea*, *Phomopsis carposchiza*, and *Diaporthe padi*) and found an inhibition percentage of up to 20%. Other previous work [86], tested two *Trichoderma* strains against 18 *Botrytis cinerea* strains and found that the mycelial growth inhibition from 74.2% to 96.9% and from 71.1% to 95.9% for *T. asperellum* and *T. harzianum*, respectively. The most inhibited pathogenic fungus as a result of *Trichoderma* strains was the *F. graminearum* compared with the other two pathogenic fungi (Table 4).

#### 3.2.4. Rate and Speed of Growth

The faster growth rates among the studied *Trichoderma* strains compared with *F. graminearum* was considered one of the most common antagonistic mechanisms as a way of competing for space and nutrients. As shown in Figure S1, *F. graminearum* was the pathogen with the slowest growth rate compared to all *Trichoderma* strains. This means that *Trichoderma* used its ability for faster growth as an essential mechanism to compete with pathogenic fungi. Moreover, rapid growth rate helps *Trichoderma* strains to compete with pathogens by solubilizing phosphate faster to facilitate their mycelial growth [8]. In comparison, most plant pathogenic fungi are incapable of solubilizing phosphate. They can easily be attacked by the highly efficient phosphate solubilizing *T. harzianum*. Several studies [20,82] reported that high temperature could induce *T. asperellum* mycelia to form a massive number of conidia in a short time and speed up the spread of conidia, forming a sizeable infected area with *Trichoderma*.

### 3.3. T. harzianum (Th6) against F. graminearum

#### 3.3.1. Mycoparasitic Activity at Different Temperatures

Different *Trichoderma* species can live in various climatic environments that determine their distributions [20]. In this experiment, the strain with the strongest inhibitory activity *T. harzianum* (*Th6*, Figure 5) was selected to inhibit *F. graminearum* at a wide range of temperatures (4 °C, 28 °C, and 37 °C), and the data are illustrated in Table S1 and (Figure 6V). The data in Table S1 indicate that *F. graminearum* grow slow rate at 4 °C than at other temperatures. Although the optimum temperature for *F. graminearum* was 25 °C, its growth rate was faster at 37 °C than 28 °C.

The highest inhibition rate of *F. graminearum* mycelial growth (76.9%) was recorded after 120 h at 37 °C, while the lowest inhibition (18%) was recorded after 72 h at 28 °C. At 28 °C and 37 °C the inhibition rates gradually increased after 72 h of growth and reached their maximum after 120 h. On the other hand, the inhibition rate of *F. graminearum* at 4 °C decreased after 72 h and then remained constant. At 4 °C, despite there is no mycelial contact between the two tested fungi, *F. graminearum* was inhibited (Figure 6 and Table S1), the reason might be related to the diffusion of secondary metabolites in the medium.

An earlier [20] clarified the effect of temperature on *Trichoderma* antagonistic activity, showing that *Trichoderma* species attach to the pathogenic fungal cell by secretion of cell wall carbohydrates which bind to the pathogenic fungi’s lectins. The lectin content of pathogenic fungi was increased at high temperature (around 36 °C), causing pathogenic fungi mycelia to adsorb more conidia and be infected by *T. asperellum*. Moreover, another study [87] demonstrated that *Trichoderma* conidia could accumulate intracellular sugars for instance mannitol and trehalose under thermal stress. These sugars could enhance both germination and conidial bioactivity as well as the tolerance of stressful conditions.

#### 3.3.2. Light Microscopic Examination

Microscopic examination of the antagonist (*T. harzianum* “*Th6*”) and pathogen (*F. graminearum*) mycelia and the interactions between them is showing in (Figure 6IV(C–F)). In this experiment, PDA plates were inoculated with two mycelial disks cut from one *Trichoderma* strain and one pathogen. The fungal strains grew toward each other, and their hyphae interacted. After 48 h of incubation at 25 °C, contact between the two fungi with parallel growth of *T. harzianum* (*Th6*) alongside *F. graminearum* hypha was spotted, then coiling of the antagonists around the pathogen were observed after 20 min (Figure 6IV(D)). Formation of appressorium-like structures and suffocation were observed after 30 and 60 min of contact (Figure 6IV(E,F)). Moreover, suffocation of *F. graminearum* mycelium was observed at contact sites with *T. harzianum*. Similar results recorded mycoparasitism (penetration, coiling and parallel growth) of *T. harzianum* against two *Colletotrichum* species [88], *Sclerotium rolfsii* [89] and *Sclerotinia sclerotiorum* [90].

## 4. Conclusions

*Trichoderma* strains could inhibit three soil-borne pathogens in vitro hyperparasitic activity. The antigenicity of endophytic fungi is not only related to the number of different species in the rhizosphere but also the diversity relationship between the different *Trichoderma* strains. This investigation showed the effectiveness of RAPD and SSR markers in ascertaining the genetic relationships among eight *Trichoderma* species. The RAPD markers proved to be less precise while the SSR markers showed the highest efficacy among the strains. The results of phosphate solubilization investigation imply that *Trichoderma* species have the ability to solubilize insoluble phosphate for enhancing the phosphate uptake by plants in fields. *T. viride* (*Tv8*) had the highest phosphate solubilization index (10.0 mm) compared with the other strains.

Additionally, *T. harzianum* (*Th6*) had the highest antagonistic activity in dual culture assay along with the growth rate. Based on our experimental approach, the *T. harzianum* (*Th6*) strain seems to present interesting features as a potential biocontrol agent. However, further studies are needed in order to determine whether the combination of the biological control and biofertilization using this strain will provide a benefit to more efficient and safer formulations to the environment and the producers. Furthermore, the analysis of secreted enzymes and secondary metabolites related to the control of the phytopathogens along with the type of plant response to this strain, would provide a better understanding between the antagonistic efficiency of *Trichoderma* strains and their diversity. In sum, this approach for the activity of microbe—microbe interactions and the multiple mechanisms of the biological agents will open up new avenues in plant beneficial microbes.

## Figures and Tables

**Table 1:** Design of species-specific β-1,3-endoglucanase, microsatellite loci and random primers used in this study for the identification of *Trichoderma* spp. and its genetic relationships

Primer Name	Primer Sequence (5′–3′)
**RAPD**	
OPA02	TGCCGAGCTG
OPA04	AATCGGGCTG
OPA05	AGGGGTCTTG
OP-A3	AGTCAGCCAC
OP-B3	CATCCCCCTG
OP-B9	TGGGGGACTC
OP-C3	GGGGGTCTTT
**SSR [42,44]**	
SSR1	F: GAAACAACACCGAAATACAC, R: CAAGTCAGATGAAGTTTG
SSR2	F: GACTCATACTTTGTTCTTAGCAG, R: GAACGGAGCGGTCACATTAG
SSR3	F: CAAGCTGACGCCTATGAAGA, R: CTTTCACTCACTCAACTCTC
SSR6	F: CCATGCATACGTGACTGC, R: GTTGACTGTTGGTGTAAGTG
SSR8	F: GGGAATTTGTGGAGGGAAG, R: CCTCAGAATGTCCCTGTC
TvCTTT29	F: GGAAGATAGCACGATGAAGTCG, R: AACCGTGGAAGTAGGTGTCG
TvCAT32	F: GTGTAGCAGCCCAACAGTCC, R: CAGGTGTCGTGACAGATTCG
β-1,3-endoglucanase	F:TCAACATCGCCAACGTCAACGAC, R: TGCCAATACGGGAACCAGTGATC

**Table 2:** RAPD and SSR-PCR analysis of *Trichoderma* strains.

Primers	Band Size (bp)	Total Number of Bands	Number of Polymorphic Bands	Polymorphic Bands Percentage (%)
RAPD				
OPA02	200–3000	9	6	66.6
OPA04	100–2500	6	4	66.6
OPA05	250–3000	9	8	88.88
OP-A3	400–1500	5	4	80
OP-B3	100–1300	5	5	100
OP-B9	200–1000	4	4	100
OP-C3	100–1300	7	5	71.4
Total	-------------	45	36	--------------
SSR				
SSR1	100–500	6	5	83.3
SSR2	100–500	5	4	80
SSR3	100–500	5	4	80
SSR6	100–500	5	4	80
SSR8	100–500	4	3	75
Tvc-29	100–400	5	5	100
Tvc-32	100–400	6	6	100
Total	-------------	36	31	---------------

**Table 3:** The growth rate of *Trichoderma* strains on different media and at different incubation temperatures.

*Trichoderma* Strains	25 °C		35 °C	
	PDA	CMD	PDA	CMD
*Ta1*	6.20 ± 0.01	4.75 ± 0.10	4.22 ± 0.02	2.60 ± 0.41
*Ta2*	5.84 ± 0.06	4.20 ± 0.08	3.20 ± 0.47	2.90 ± 0.18
*Th3*	5.10 ± 0.01	5.10 ± 0.29	2.75 ± 0.38	3.50 ± 0.38
*Th4*	5.33 ± 0.09	4.10 ± 0.13	2.50 ± 0.31	3.20 ± 0.09
*Tl5*	6.10 ± 0.44	4.90 ± 0.50	3.90 ± 0.44	2.75 ± 0.46
*Th6*	6.00 ± 0.47	5.30 ± 0.19	4.30 ± 0.13	3.80 ± 0.56
*Th7*	5.95 ± 0.14	5.25 ± 0.14	3.50 ± 0.89	3.00 ± 0.67
*Tv8*	5.00 ± 0.45	4.88 ± 0.72	2.70 ± 0.57	2.30 ± 0.15

CMD = cornmeal dextrose agar; PDA = potato dextrose agar. Data are means ± Standard error.

**Table 4:** Antagonistic activity of *Trichoderma* strains against three pathogenic fungi by dual culture technique.

*Trichoderma* Strains	*F. solani*		*M. phaseolina*		*F. graminearum*	
	RMG (cm)	IMG (%)	RMG (cm)	IMG (%)	RMG (cm)	IMG (%)
Control	3.50 ± 0.06	0.00	3.70 ± 0.30	0.00	6.50 ± 0.16	0.00
*Ta1*	1.40 ± 0.09	60.00	1.20 ± 0.31	67.56	1.50 ± 0.08	76.92
*Ta2*	1.65 ± 0.06	52.85	1.10 ± 0.10	70.27	1.20 ± 0.21	81.53
*Th3*	1.55 ± 0.15	55.71	1.30 ± 0.22	64.86	1.50 ± 0.17	76.92
*Th4*	1.75 ± 0.38	50.01	1.14 ± 0.07	69.18	1.80 ± 0.49	72.30
*Tl5*	1.70 ± 0.32	51.42	1.01 ± 0.11	72.96	1.20 ± 0.01	81.53
*Th6*	1.00 ± 0.01	71.42	1.00 ± 0.13	72.97	1.00 ± 0.15	84.61
*Th7*	1.55 ± 0.40	55.71	1.20 ± 0.20	67.56	1.01 ± 0.01	84.60
*Tv8*	1.75 ± 0.30	50.00	1.33 ± 0.08	64.05	2.00 ± 0.32	69.23

RMG: Radial Mycelial growth; IMG: Inhibition of mycelial growth. Data are means ± Standard error.

## Supplementary Materials

The following are available online at https://www.mdpi.com/2079-7737/9/8/189/s1, Figure S1: Competition of fungal pathogen *F.g* and *Trichoderma* strains, Table S1: Antagonistic activity of *T. harzianum* (*Th6*) against *F. graminearum* under thermal stress.
