# Supplementary material for: Phylogenetic Diversity of Trichoderma Strains and Their Antagonistic Potential against Soil-Borne Pathogens under Stress Conditions
Source: Biology (Basel). 2020 Jul 23;9(8):189. doi: 10.3390/biology9080189 (PMC7466124; doi:10.3390/biology9080189)
Supplement: Supplementary file 1 [file biology-09-00189-s001.pdf]

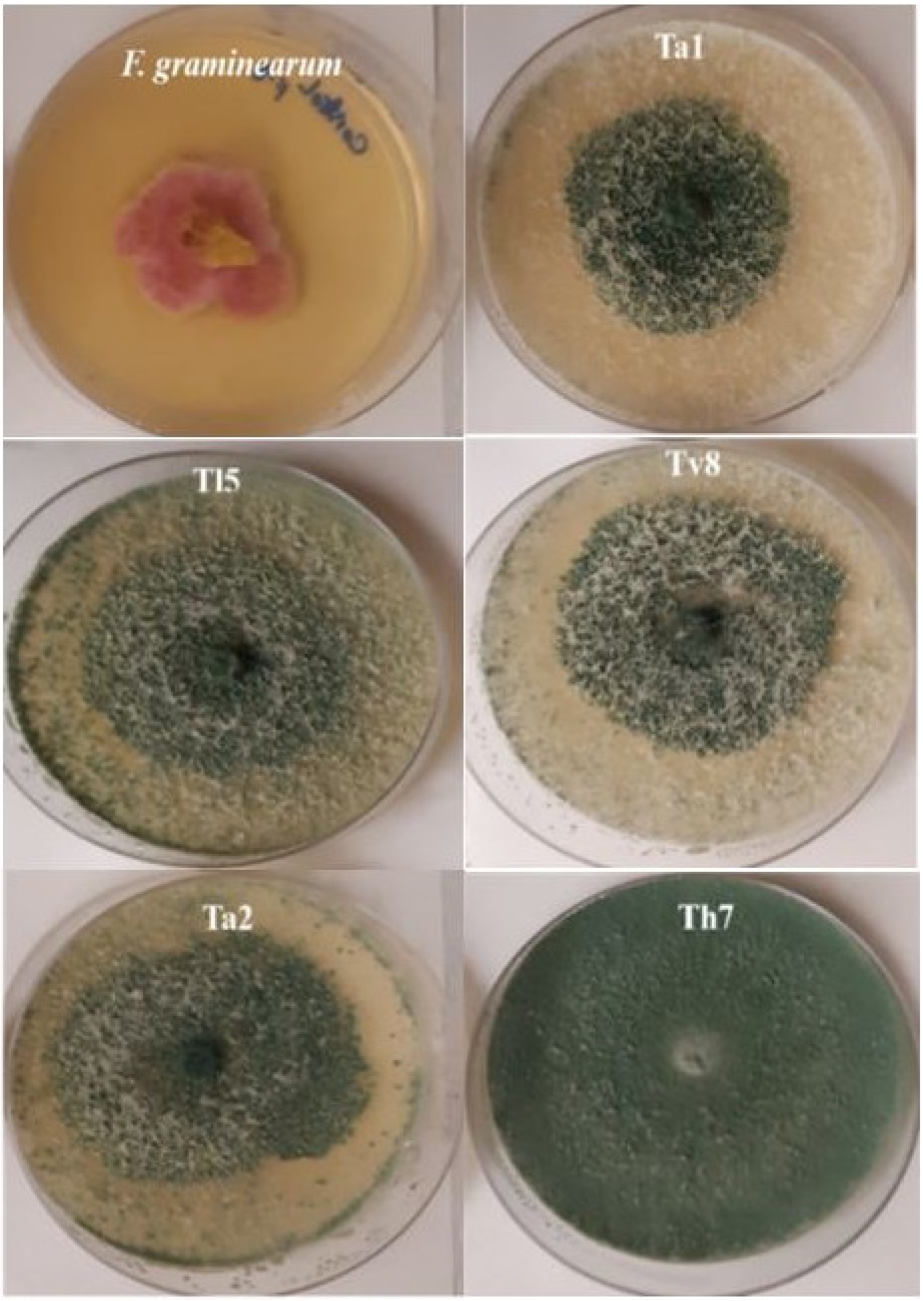

Figure S1. Competition of fungal pathogen *F.g* and *Trichoderma* strains.

Table S1. Antagonistic activity of *T. harzianum* (Th6) against *F. graminearum* under thermal stress.

**Mycelial growth (mm)**	**4 °C**			**28 °C**			**37 °C**		
	**72 h**	**96 h**	**120 h**	**72 h**	**96 h**	**120 h**	**72 h**	**96 h**	**120 h**
F. graminearum	0.53	0.63	0.63	1.70	1.87	2.37	2.20	2.37	3.60
F. graminearum + Th6	0.40	0.50	0.50	1.33	1.13	0.90	1.40	1.10	0.80
Inhibition rate (%)	24.3	20.6	20.6	18.0	39.7	62.1	36.0	55.0	76.9
